# Book Review

**DOI:** 10.1054/bjoc.1999.0910

**Published:** 1999-12-08


*British Journal of Cancer* (2000) **82**(1), 250
© 2000 Cancer Research Campaign
Article no. bjoc.1999.0910

### Book Review

### The surgery of childhood tumors

Edited by R Carachi, A Azmy and JL Grosfeld, pp., £175.00, ISBN

The dramatic improvement in survival of children with cancer has been one of the success stories of paediatric oncology over the past two to three decades. Although the greatest improvements in survival have been achieved for leukaemia and lymphoma, there have been spectacular advances in the management of solid tumours in recent years. Currently, the expected overall survival rate for nephroblastoma (Wilms' tumour) should approach 85%, germ-cell tumours 90%, sarcomas 60% and hepatoblastoma 70–80%, while survival for neuroblastoma lags behind at 40% overall, with the greatest improvement being in Stage 3 disease. During this period, the role of the surgeon has changed from being 'in-charge' to that of a member of a multidisciplinary team and most patients nowadays are referred primarily to the paediatric oncologist.

The editors of this book have gathered together in a single volume a wide spectrum of subjects on solid tumours in children produced by a large number of contributors all of whom are experts in the field. The first section of the book comprises seven chapters on the generality of childhood tumours. The 5-year survival for a range of malignancies in children in the UK provides statistics up to 1990. Since then there has been a notable increase in survival for both acute non-lymphoblastic leukaemia and Ewing's sarcoma but progress for neuroblastoma, despite intensified therapy, remains disappointing. There follows excellent chapters on tumour biology, imaging and pathology. A notable omission in this section is a chapter on the insertion, care and complications of central venous catheters. This procedure is currently the most common operation carried out by paediatric surgeons and/or interventional radiologists and therefore merits inclusion.

Part B comprises seven chapters concerning tumours most frequently managed by paediatric surgeons. There is a good comprehensive chapter on neonatal tumours. The chapter on nephroblastoma does not include any details on the chemotherapy regimens currently employed nor does it discuss the controversy regarding immediate surgery versus preoperative chemotherapy followed by delayed resection. Neuroblastoma is covered well with a useful and comprehensive up-to-date selection of references. The staging system is repeated elsewhere in the book and would have been better referenced only in later sections.

The final part comprises chapters on tumours some of which are managed only by specialist surgeons, i.e. bone and brain tumours. Repetition again features in the chapter on thoracic tumours and the chapter on rare tumours comprises only brief statements on a wide variety of tumours, and is of limited value. Reconstructive surgery is largely limited to urinary bladder reconstruction which is covered in great technical detail.

Complications associated with the treatment of childhood malignancy are, unfortunately, not infrequent. Necrotizing fasciitis must be treated early by aggressive excision if the child is to be given any chance of survival. The acute abdomen may pose major diagnostic difficulties and should be thoroughly investigated as it would be a tragedy for a child in remission on chemotherapy to suffer from delayed diagnosis of acute appendicitis or adhesive intestinal obstruction.

An increasingly important aspect in the management of childhood malignancy is supportive therapy and pain management and palliative care and this is appropriately discussed in the final chapter.

On the whole, I would recommend this book to general paediatric surgeons with an interest in oncology. It is, however, comparatively quite expensive, £175.00, but all paediatric libraries should acquire a copy.

*L Spitz*
*Paediatric Surgical Unit,*
*Institute of Child Health,*
*30 Guildford Street,*
*London WC1N 1EH, UK*

